# The arthroscopical and radiological corelation of lever sign test for the diagnosis of anterior cruciate ligament rupture

**DOI:** 10.1186/s40064-015-1628-9

**Published:** 2015-12-30

**Authors:** Alper Deveci, Deniz Cankaya, Serdar Yilmaz, Güzelali Özdemir, Emrah Arslantaş, Murat Bozkurt

**Affiliations:** Department of Orthopaedics and Traumatology, Ankara Numune Training and Research Hospital, Ankara, Turkey; Department of Orthopaedics and Traumatology, Fatih Sultan Mehmet Training and Research Hospital, Istanbul, Turkey; School of Medicine, Department of Orthopaedics and Traumatology, Yildirim Beyazıt University, Ankara, Turkey; Turgut Ozal Mahallesi 2141, Sokak Akkent 2 Sitesi, B Blok 36, Batıkent/Ankara, Turkey

## Abstract

The aim of the current study was to evaluate the sensitivity of the lever sign test and the widely used basic tests of the Lachman, anterior drawer and pivot shift tests, both under anaesthesia and without anaesthesia, according to the gold standard diagnostic arthroscopic results in patients undergoing anterior cruciate ligament reconstruction. The study included 117 patients, diagnosed with ACL tear which was definitively determined during an arthroscopic surgical procedure applied. Before anaesthesia and while under anaesthesia, the Lachman, anterior drawer, pivot shift and lever sign tests were applied to all patients. Evaluation was made of MR images for each patient and documented. The patients comprised 96 males and 21 females, witha mean age of 25.8 ± 5.9 years (range, 17–45 years). Total tear was determined in 82 cases, anteromedial (AM) bundle in 14, posterolateral (PL) bundle in 13 and elongation in 8. Pre-anaesthesia positivity was found in lever sign at 94.2 %, Lachman at 80.5 %, pivot shift at 62.3 % and anterior drawer at 60.1 %. These rates were determined after anaesthesia as lever sign 98.4 %, Lachman 88.7 %, pivot shift 88.3 % and anterior drawer 84.2 %. The lever sign test can be easily applied clinically and it seems to have higher sensitivity than the Lachman test which is the basis of classic information, it should be included in routine clinical practice. In the light of the results of this study, further studies are required to review the accepted view that the Lachmann test is the most reliable test.

## Background

Anterior cruciate ligament (ACL) injuries are the most frequently seen ligament injuries of the knee joint (Benjaminse et al. 2006; Prodromos et al. 2007). Diagnosis is based on history, physical examination and MRI findings and a definitive diagnosis is confirmed with arthroscopic imaging (Crawford et al. 2007; Oei et al. 2003; Finsterbush et al. 1989; Farquharson-Roberts and Osborne 1983; McDaniel 1976; Noyes et al. 1989; Lintner et al. 1995; DeFranco and Bach 2009). Although arthroscopic evaluation is the gold standard, the tear cannot be determined even with this method in cases where there is incorrect evaluation of the femoral attachment site and when there is continued integrity in the ACL fibres (DeFranco and Bach 2009).

In the clinical evaluation, the first and most important step of the patient history is the physical examination. Three basic tests are used in the physical examination from which different results may be obtained according to the sensitivity and specificity of each test. These are the Lachman, the anterior drawer and the pivot shift test (DeFranco and Bach 2009; Johannsen and Fruensgaard 1988; van Eck et al. 2013). Generally there are two problems related to physical examination methods. In partial tears in particular, in contrast to the complaint of instability, the findings of the physical examination made with the three tests in question may be normal (Lintner et al. 1995; DeFranco and Bach 2009; Zantop et al. 2007). The other problem is that the patient may have developed pain-resistance (Benjaminse et al. 2006; van Eck et al. 2013). In these situations, MRI evaluation is presented as a choice for making a diagnosis. Although MRI has reliability of 94–98 %, it is not practical, is expensive and should not be valued as a stand-alone test (Yao et al. 1995; Umans et al. 1995; Hong et al. 2003; Friedman and Jackson 1996; Kelly et al. 1991). Therefore, it should be combined in an approach with an efficient physical examination (Meuffels et al. 2012; Liu et al. 1995; Gelb et al. 1996; Kocabey et al. 2004). Despite the negative aspects, the Lachman, the anterior drawer and the pivot shift test are the most significant stage of the diagnostic approach as they can be applied easily, are cheap and non-invasive. Several evaluation tests have been developed to overcome the drawbacks. One of these, developed in recent years by A. Lelli is the ‘lever sign’ test (Lelli et al. 2014). It has been claimed that this test is more valuable than the other 3 tests in both partial and total lesions. In particular, it has been suggested that it could be applied effectively, regardless of the interval from trauma to examination. In literature, there is only the study by Alessandro Lelli related to the lever sign test, in which the sensitivity of the lever sign test and the sensitivity of the three basic tests was evaluated according to the MRI results without using the gold standard arthroscopic evaluation criteria.

The aim of the current study was to evaluate the sensitivity of the lever sign test and the widely used basic tests of the Lachman, anterior drawer and pivot shift tests, both under anaesthesia and without anaesthesia, according to the gold standard diagnostic arthroscopic results with reference to the results of diagnostic arthroscopy in patients undergoing anterior cruciate ligament reconstruction. The hypothesis of the study was that the lever sign test had higher sensitivity than the other three tests and was less affected by patient-related factors in patients under anaesthesia and without anaesthesia.

## Methods

Approval for the study was granted by the Institutional Review Board. The study included 117 patients, diagnosed with ACL tear which was definitively determined during an arthroscopic surgical procedure applied between January–August 2015. The decision for surgical intervention was made by a combined evaluation of the physical examination, MRI and the patient’s complaints of instability. For patients found to have an intact ACL in the physical examination and MRI evaluation, the decision for an arthroscopic intervention was made according to complaints of instability such as the feeling of giving way, unreliability, pain and weakness. All examinations were performed on a 1.5 T whole body MRI system (Excite, General Electrics, Milwaukee, Wisconsin) with a 33 mT/m maximum gradient capacity.

Following the trauma, all patients were given cold pack therapy, anti-inflammatory medical treatment and weight-bearing was not permitted in the first week. Before the arthroscopic procedure, quadriceps and hamstring strengthening exercises were applied for at least 3 weeks. The mean period between trauma and surgery was 8.7 weeks (range, 4–25 weeks). All patients were admitted for surgery under spinal anaesthesia without the application of muscle relaxant. The cases for which ACL reconstruction procedures were applied after the 4th week were grouped as acute or chronic. Patients were excluded if there was medial meniscus posterior root tear, bilateral ACL tear, multiple ligament injuries or previous arthroscopic surgery.

**Fig. 1:**
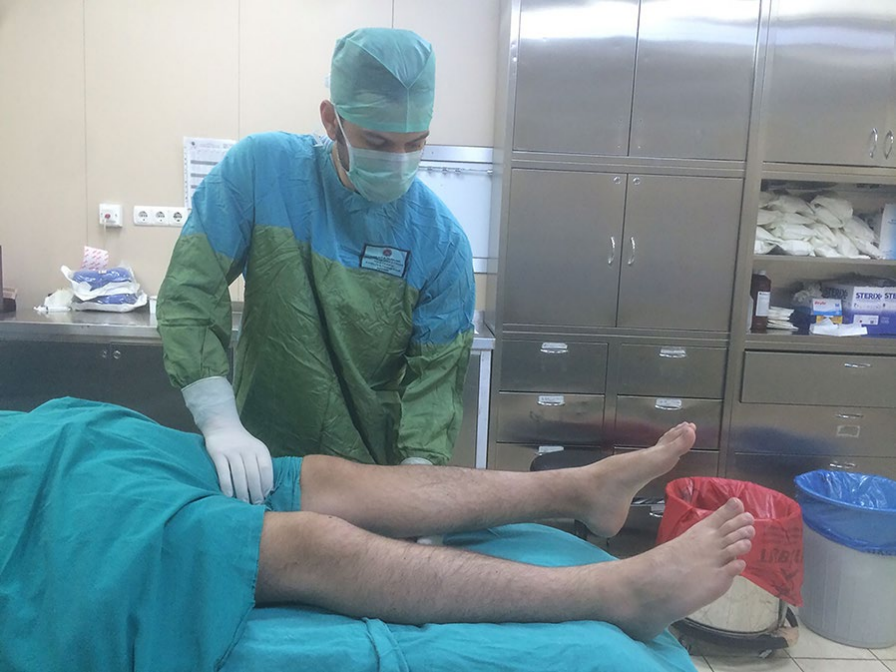
The view of the intact ACL side. If the femoral condyle moves to the posterior, the proximal tibia will move to the anterior

**Fig. 2:**
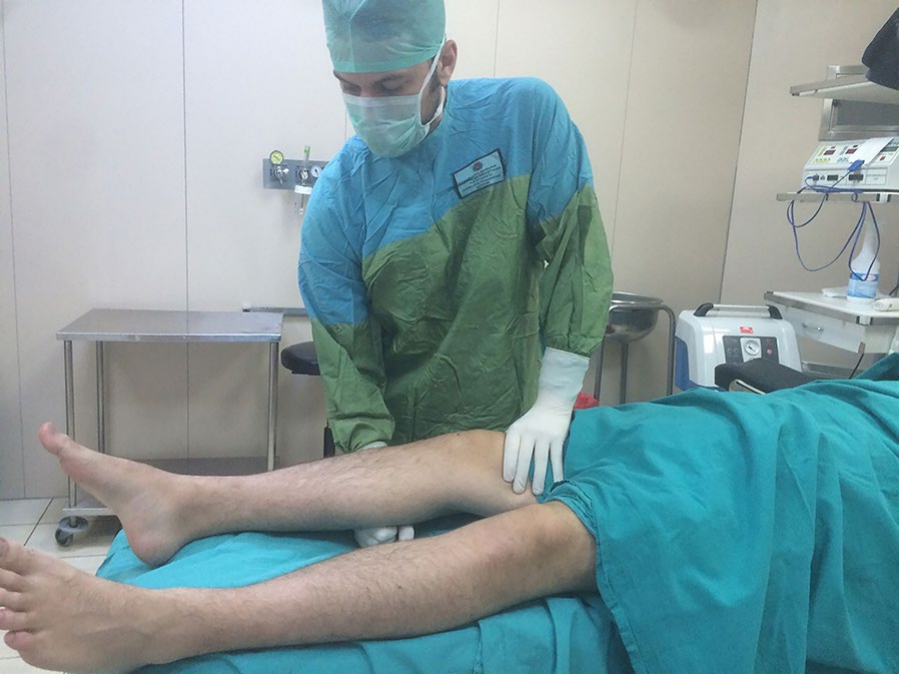
The view of the injured ACL side. The downward pressure of the movement of the distal femur will not be followed by proximal tibia movement and the heel will not be able to be raised

The lever sign test was applied as described by Lelli (2014). The patient was positioned supine on the operating table by the clinician with both legs extended and the side of the ACL lesion was determined. If it was on the patient’s left side, the clinician placed his left hand as a fist below the proximal third of the cruris and if on the right side, the right hand as a fist. Thus, with the knee brought slightly into flexion, the heel made contact with the operating table. With the other hand, force was applied over the distal third of the quadriceps downwards onto the thigh. The hand as a fist forms a point of resistance and thereby 2 force vectors are created. One is the clinician’s hand pressing down on the quadriceps and the other is the gravitational force of the lever applied on the other side to the leg and foot. With an intact ACL the proximal tibia will move anteriorly when the femoral condyle is pushed posteriorly. An upward movement will be formed in the foot and leg distal of the hand functioning as support (Fig. 1). When the ACL is damaged, the downward pressure of the movement of the distal femur will not be followed by proximal tibia movement and the tibia will remain more anterior than the femur. As a result the heel will not rise, which shows that the test is positive (Fig. 2). All the patients were examined by two clinicians (AD, DC) to assess the inter-rater reliability and each clinician was blinded to the assessment of the other during the physical examinations. The test has been applied to both the injured and uninjured side. Evaluation was made of MR images for each patient and documented.

### Statistical analysis

Descriptive statistics were used to characterize the sample. Sensitivity was calculated by measuring the proportion of actual positives in the total sample and formulated as true positives/(true positive + false negative). All calculations were performed with SPSS 20.0 software (SPSS Inc.,Chicago, IL, USA.).

## Results

The patients comprised 96 males and 21 females, with a mean age of 25.8 ± 5.9 years (range, 17–45 years) Table 1. Total tear was determined in 82 cases, anteromedial (AM) bundle in 14, posterolateral (PL) bundle in 13 and elongation in 8. Pre-anaesthesia positivity was found in lever sign at 94.2 %, Lachman at 80.5 %, pivot shift at 62.3 % and anterior drawer at 60.1 %. These rates were determined after anaesthesia as lever sign 98.4 %, Lachman 88.7 %, pivot shift 88.3 % and anterior drawer 84.2 %. The sensitivity values of the lever sign, Lachman, pivot shift and anterior drawer tests at pre-anaesthesia and under anaesthesia are summarised in Table 2. The intra-class correlation (ICC) was used to assess inter-rater reliability (IRR). Any measurement with a reliability coefficient of >0.75 was considered to have good reliability (Portney and Watkins 2000). All the ICC values (0.89–0.96 for lever sign test, 0.85–0.91 for Lachman test, 0.82–0.88 for pivot shift test and 0.84–0.93 for anterior drawer test) for the physical examination tests were >0.75 in the present study. Therefore, the physical examination data of the study were accepted as good and reliable.

**Table 1:** Demographic data of the patients

Gender (n)	
Female	21
Male	96
Age (years)	25.8 ± 5.9 (17–45)
BMI (kg/m^2^)	23.95 (21.90–26.80)

*BMI* body mass index

**Table 2:** The sensitivity values of stress tests at pre- anaesthesia and under anaesthesia

	Pre-anaesthesia assesment (%)	Under anaesthesia assesment (%)
Lever sign test	94	98
Lachman test	80	88
Pivot shift test	62	88
Anterior drawer test	60	88

MRI sensitivity was determined as 92.3 %. In 9 patients determined with total ACL tear in the arthroscopic evaluation, it was reported as intact in the MRI evaluation.

According to these results, the lever sign test has much higher sensitivity than the other tests both before anaesthesia and under anaesthia. The sensitivity value under anaesthesia was even found to be higher than the MRI evaluation. In the pre-anaesthesia evaluation, the examination method with the lowest reliability was seen to be the anterior drawer test. The test with the lowest sensitivity under anaesthesia was determined to be the pivot shift test.

## Discussion

The most significant finding of this study was that the lever sign test is a new test with higher sensitivity than the Lachman test which can be applied easily both under anaesthesia and without anaesthesia.

Due to the stress of the trauma and patient resistance, it may be difficult to apply the standard physical examination methods effectively. Secondly, if the tear is partial or if the ruptured ACL is attached to another point, false negative results may be given (Lintner et al. 1995; DeFranco and Bach 2009; van Eck et al. 2013; Zantop et al. 2007; Katz and Fingeroth 1986). Different results may also be obtained depending on the person applying the examination and whether or not the examination method is applied under anaesthesia (van Eck et al. 2013). The lever sign test differs from other tests in that the basic area of manipulation is primarily not the tibia but the femur (Lelli et al. 2014). Furthermore, application of the test is very easy and practical. There is no difficult learning curve for the test.

As 85 % of ACL injuries are from the femoral attachment and there can be considered to be a possibility of attachment of the ruptured ligament to the PCL or structures within the femoral notch, it is more correct for manipulation to be made from the femur.

In the study by Lelli, it was determined that the lever sign test had high sensitivity regardless of the degree of the ACL tear or the time of injury (Lelli et al. 2014). In that study, the lever sign test results were consistent with the MRI examination. However, because of errors in application technique or evaluation of MRI, false negative results may be obtained (Liu et al. 1995; Gelb et al. 1996; Kocabey et al. 2004; Steckel et al. 2006). The decision for an arthroscopic approach was based on complaints of instability in the current cases and patients were included for whom ACL reconstruction was planned. As arthroscopic evaluation is the gold standard in the diagnosis of ACL tear, the test was truly evaluated. Thus it was thought that more accurate results were obtained from a point confirming the definite ACL injury.

In literature, the Lachman test has been reported as the test with the highest sensitivity and the pivot shift as that with the highest specificity (Benjaminse et al. 2006; DeFranco and Bach 2009; van Eck et al. 2013; Steckel et al. 2006). Lelli et al. evaluated the lever sign, Lachman, anterior drawer and pivot shift tests. Sensitivity rates were found to be 1.00, 0.62, 0.72 and 0.47 respectively. Sensitivity of the Lachman test was seen to be lower than rates in literature and in the current study (Lelli et al. 2014). Pre-anaesthesia sensitivity in the current study was determined as lever sign 0.94, Lachman 0.80, anterior drawer 0.60 and pivot shift test 0.62. Under anaesthesia, these values were determined as lever sign 0.98, Lachman 0.94, anterior drawer 0.84 and pivot shift test 0.78. The sensitivity of the lever sign test was extremely high when applied both before and under anaesthesia and the results were seen to be consistent with those of the study by Lelli.

The high sensitivity value of the Lachman test in the current study may be due to low patient resistance as there was no acute trauma group. The lever sign test was seen to have much higher sensitivity compared to the other tests in both the evaluation pre-anaesthesia and under anaesthesia. Under anaesthesia, an increase was seen in the sensitivity of the lever sign test and of the other tests. Although this change was significant, it was not at a very high rate in the lever sign test. This shows that the test was affected less than the other tests by patient-related factors.

In a meta-analysis by Carola F. Van Eck et al., the sensitivity of the Lachman, anterior drawer and pivot shift tests applied without anaesthetic was found to be 0.81, 0.38 and 0.28 respectively (van Eck et al. 2013). In the current study, the pre-anaesthesia sensitivity values were determined as Lachman 0.80, anterior drawer 0.60, pivot shift 0.62 and lever sign test 0.94. In contrast to the similarity found in the sensitivity of the Lachman test, the sensitivity of the pivot shift test and anterior drawer test in the current study was found to be higher than that of the study by Carola F. Van Eck. This may be due to low patient resistance as there was no acute trauma group in the current study. In the meta-analysis by Carola F. Van Eck et al., the sensitivity values of the Lachman, anterior drawer and pivot shift tests applied under anaesthetic were found to be 0.91, 0.63 and 0.73 respectively. A significant increase was seen in the sensitivity of all 3 tests under anaesthesia, showing that all 3 tests were affected by patient-related factors.

In the Lelli study, the sensitivity of the examination method was evaluated according to the MRI diagnosis of the ACL tear (Lelli et al. 2014). The sensitivity of MRI in the evaluation of ACL tear has been reported as 92–96 % (Steckel et al. 2006; Lefevre et al. 2014). Although arthroscopic evaluation is an invasive method, it is the gold standard for definitive diagnosis (Crawford et al. 2007; Oei et al. 2003). A superior aspect of the current study is that the diagnosis of ACL tear and type were clearly established. Thus, by referring back to a definite diagnosis, the accuracy of the examination method was better checked. The high values obtained in the the lever sign test both before anaesthesia and under anaesthesia showed that even if there were external effects such as pain and patient resistance, the accuracy rate in the current study cases, including partial tears, was much higher.

A limitation of the current study was the small sample size as the evaluation was only of arthroscopic surgery cases. For the same reason, no acute cases were evaluated and therefore the study only included chronic cases. As the number of cases with partial tear was extremely low, test reliability could not be evaluated in respect of partial and full tear cases. Evaluation was not made according to different types of anaesthesia. As the number of females in the sample was low, differences in terms of gender were not evaluated. Finally, when evaluating the sensitivity of the tests, no examination was made of specificity.

Diagnosis of ACL tear is made by a combined evaluation of the patient history, physical examination and MRI. Accurate diagnosis is made by interpretation of these 3 steps together. The physical examination methods are indispensable as practical, cheap, non-invasive methods.

## Conclusion

The lever sign test can be easily applied clinically and it seems to have higher sensitivity than the Lachman test which is the basis of classic information, it should be included in routine clinical practice. In the light of the results of this study, further studies are required to review the accepted view that the Lachmann test is the most reliable test.
